# Tetra­kis(μ-4-*tert*-butyl­benzoato)-κ^3^ *O*:*O*,*O*′;κ^3^ *O*,*O*′:*O*′;κ^4^ *O*:*O*′-bis­[aqua­bis­(4-*tert*-butyl­benzoato-κ^2^ *O*,*O*′)(4-*tert*-butyl­benzoic acid-κ*O*)praseodymium(III)]

**DOI:** 10.1107/S1600536811029667

**Published:** 2011-07-30

**Authors:** Jun Dai, Rong-Kun Pan, Juan Yang

**Affiliations:** a Institute of Safety Science and Engineering, Henan Polytechnic University, Jiaozuo 454003, People’s Republic of China; b Department of Physics and Chemistry, Henan Polytechnic University, Jiaozuo 454003, People’s Republic of China

## Abstract

The reaction of praseodymium nitrate and 4-*tert*-butyl­benzoic acid (*t*BBAH) in aqueous solution yielded the dinuclear title complex, [Pr_2_(C_11_H_13_O_2_)_6_(C_11_H_14_O_2_)_2_(H_2_O)_2_], which has non-crystallographic *C_i_* symmetry. The two Pr^III^ ions are linked by two bridging and two bridging–chelating *t*BBA ligands, with a Pr⋯Pr separation of 4.0817 (9) Å. Each Pr^III^ ion is nine-coordinated by one chelating *t*BBA ion, one monodentate *t*BBAH ligand and one water mol­ecule in a distorted tricapped trigonal–prismatic environment. The complex mol­ecules are linked into infinite chains along the *c* axis by inter­molecular O—H⋯O hydrogen bonds.

## Related literature

For the structures and properties of lanthanide benzoate complexes, see: Roh *et al.* (2005 ▶); Singh *et al.* (2007 ▶); Xu *et al.* (2009 ▶); Yang *et al.* (2010 ▶). For bond lengths and angles in other complexes with nine-coordinated Pr^III^, see: Li *et al.* (2007 ▶); Yang *et al.* (2009 ▶).
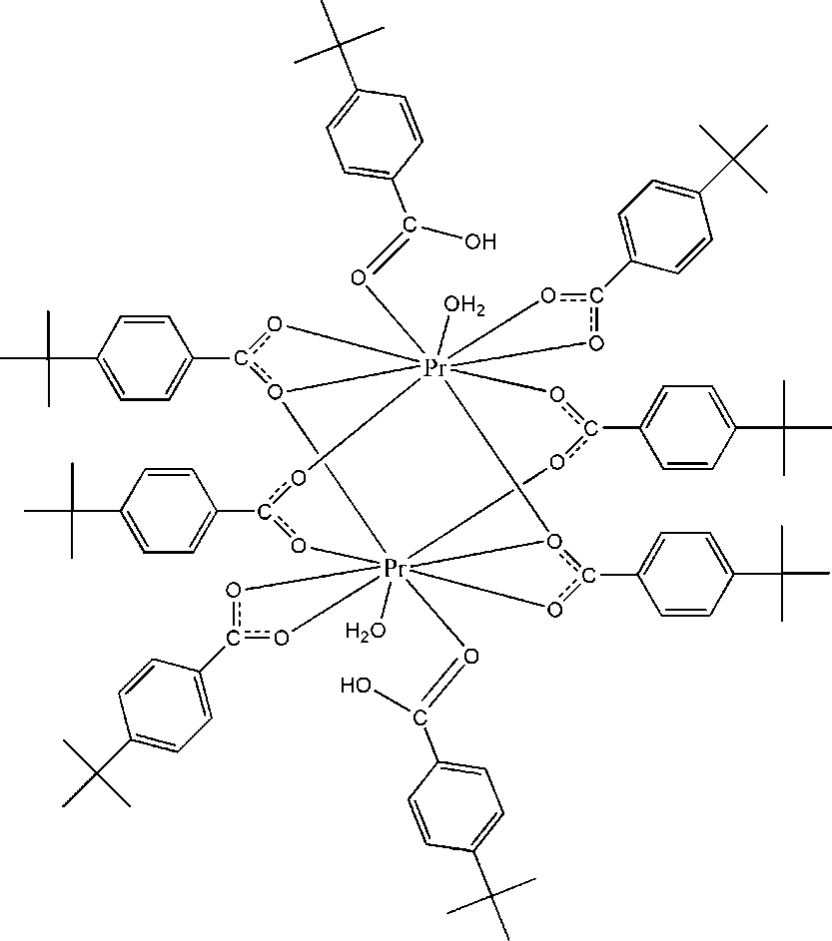


## Experimental

### 

#### Crystal data

[Pr_2_(C_11_H_13_O_2_)_6_(C_11_H_14_O_2_)_2_(H_2_O)_2_]
*M* *_r_* = 1737.58
Monoclinic, 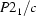

*a* = 35.194 (6) Å
*b* = 9.3857 (18) Å
*c* = 27.956 (5) Å
β = 107.073 (4)°
*V* = 8827 (3) Å^3^
*Z* = 4
Mo *K*α radiation
μ = 1.15 mm^−1^
*T* = 293 K
0.24 × 0.09 × 0.05 mm

#### Data collection

Bruker APEXII CCD area-detector diffractometer
Absorption correction: multi-scan (*SADABS*; Bruker, 2007 ▶) *T*_min_ = 0.769, *T*_max_ = 0.841
44864 measured reflections
15556 independent reflections
10611 reflections with *I* > 2σ(*I*)
*R*_int_ = 0.073

#### Refinement

*R*[*F* ^2^ > 2σ(*F* ^2^)] = 0.073
*wR*(*F* ^2^) = 0.206
*S* = 1.02
15556 reflections
999 parameters
1092 restraints
H-atom parameters constrained
Δρ_max_ = 5.46 e Å^−3^
Δρ_min_ = −1.00 e Å^−3^

### 

Data collection: *APEX2* (Bruker, 2007 ▶); cell refinement: *SAINT* (Bruker, 2007 ▶); data reduction: *SAINT*; program(s) used to solve structure: *SHELXS97* (Sheldrick, 2008 ▶); program(s) used to refine structure: *SHELXL97* (Sheldrick, 2008 ▶); molecular graphics: *SHELXTL* (Sheldrick, 2008 ▶); software used to prepare material for publication: *SHELXTL*.

## Supplementary Material

Crystal structure: contains datablock(s) global, I. DOI: 10.1107/S1600536811029667/hg5053sup1.cif

Structure factors: contains datablock(s) I. DOI: 10.1107/S1600536811029667/hg5053Isup2.hkl

Additional supplementary materials:  crystallographic information; 3D view; checkCIF report

## Figures and Tables

**Table 1:** Hydrogen-bond geometry (Å, °)

*D*—H⋯*A*	*D*—H	H⋯*A*	*D*⋯*A*	*D*—H⋯*A*
O2*W*—H2*WB*⋯O4^i^	0.82	2.25	2.824 (8)	127
O2*W*—H2*WA*⋯O5^i^	0.82	1.95	2.759 (8)	167
O1*W*—H1*WA*⋯O13^ii^	0.82	2.50	2.866 (8)	108
O1*W*—H1*WB*⋯O10^ii^	0.82	2.01	2.767 (8)	154

Symmetry codes: (i) 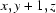; (ii) 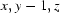.

**Fig. 1.**
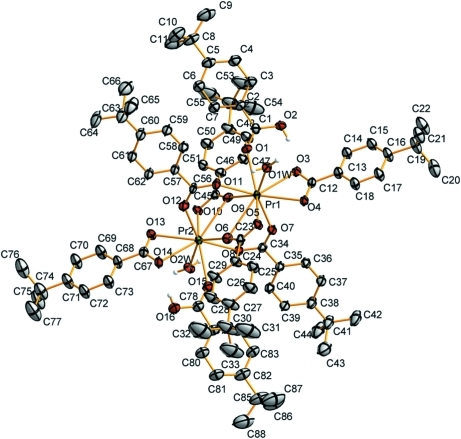
The molecular structure of the title complex, showing the atom-labeling scheme. Displacement ellipsoids for non-hydrogen atoms are drawn at the 30% probability level. All the aryl and methyl type H-atoms have been omitted for clarity.

**Fig. 2.**
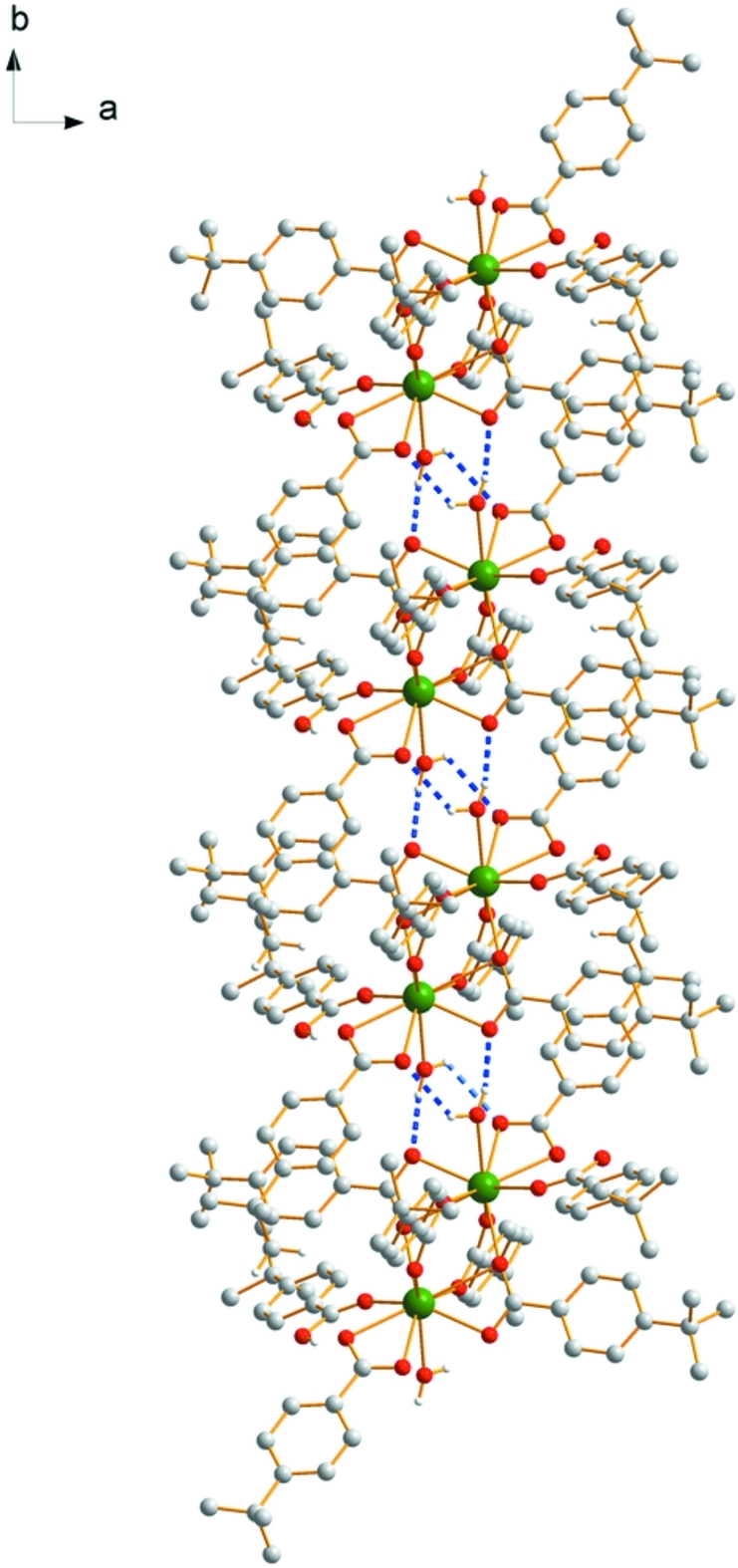
The one-dimensional-chain structure of the title compound, viewed along the c axis. Intermolecular hydrogen bonds are shown as dashed lines in blue.

## supplementary crystallographic information

### Comment

Lanthanide carboxylate complexes show interesting crystal structures due to the variable coordination number of metal centers as well as coordination versatility of carboxylate ligands. As rigid ligands, benzoic acid and its derivatives have been widely used for lanthanide complexes because they improve the thermal stability and luminescence (Roh *et al.*, 2005; Singh *et al.*, 2007; Xu *et al.*, 2009). In continuation to our research (Yang *et al.*, 2010), we now report the preparation and crystal structure of a new Pr^III^ complex obtained in the reaction with 4-*tert*-butyl-benzoic acid (*t*BBAH).

The asymmetric unit of the title dinuclear complex (Fig.1) contains two Pr atoms, six *t*BBA ligands, two *t*BBAH ligands and two coordinated water molecules. The title molecule [Pr_2_(C_11_H_13_O_2_)_6_(C_11_H_14_O_2_)_2_(H_2_O)_2_] has a non-crystallographic *C_i_* symmetry. The *t*BBA ligands show four coordination modes: bridging bidentate, chelating bidentate, bridging-chelating tridentate and monodentate mode (*t*BBAH). The two Pr^III^ ions are linked by four bridging *t*BBA ligands, with a Pr···Pr seperation of 4.0817 (9) Å. Each Pr^III^ ion is additionally coordinated by one *O*,*O*'-bidentate *t*BBA ion, one monodentate *t*BBAH ligand and one water molecule. The Pr—O bond lengths are in the range 2.395 (6)-2.807 (6) Å, which is comparable to those reported for other Pr complexes with oxygen environment around the central metal (Li *et al.*, 2007; Yang *et al.*, 2009). Each nine-coordinated Pr^III^ ion adopts a distorted tricapped trigonal-prismatic geometry. The crystal packing is stabilized by intermolecular O—H···O hydrogen bonds (Table 2), which link the molecules into one-dimensional chains along the *c* axis (Fig.2).

### Experimental

A mixture of Pr(NO_3_)_3_ 6H_2_O (0.226 g, 0.52 mmol), *t*BBAH (0.150 g, 0.84mmol), melamine (0.026 g, 0.20 mmol) and distilled water (10 ml) was sealed in a 25 ml Teflon-lined stainless autoclave. The mixture was heated at 423 K for 5 days to give the green prism crystals suitable for X-ray diffraction analysis.

M.p.:510.0-511.5 K; Anal. Calcd. for C_88_H_110_O_18_Pr_2_ (%): C, 60.83; H, 6.34; Pr, 16.24. Found (%): C, 60.62; H, 6.48; Pr, 15.97; Powder X-ray diffraction (PXRD) data are in good agreement with that simulated from the single crystal diffraction data, indicating the high phase purity of as-synthesized. IR (KBr pellet, cm^-1^): 3433 (νO-H)(br), 2964-2869 (νC-H)(m),1689(νCO(-COOH)(m), 1599 (νC=C)(s), 1550 (νas,-COO)(s), 1399 (νs,-COO)(s), 539 (νPr-O)(w).

### Refinement

All H atoms bound to C atoms were placed in calculated positions and treated in a riding-model approxination, with C—H = 0.93Å and 0.96Å for aryl and methyl type H-atoms, respectively with U_iso_ = 1.2U_eq_(C) or 1.5 U_eq_(C). The H-atoms of water molecules were located from difference maps and treated as riding with an idealized distance of O—H = 0.82 Å and U_iso_(H) = 1.5U_eq_(O). The position of carboxylic H atoms were also located in calculated positions and treated in a riding-model approxination with O-H = 0.82 Å and U_iso_(H) = 1.5U_eq_(O).

### Crystal data

**Table:**

[Pr_2_(C_11_H_13_O_2_)_6_(C_11_H_14_O_2_)_2_(H_2_O)_2_]	*F*(000) = 3600
*M**_r_* = 1737.58	*D*_x_ = 1.307 Mg m^−^^3^
Monoclinic, *P*2_1_/*c*	Mo *K*α radiation, λ = 0.71073 Å
Hall symbol: -P 2ybc	Cell parameters from 5280 reflections
*a* = 35.194 (6) Å	θ = 2.3–21.5°
*b* = 9.3857 (18) Å	µ = 1.15 mm^−^^1^
*c* = 27.956 (5) Å	*T* = 293 K
β = 107.073 (4)°	Prism, green
*V* = 8827 (3) Å^3^	0.24 × 0.09 × 0.05 mm
*Z* = 4	

### Data collection

**Table:**

Bruker APEXII CCD area-detector diffractometer	15556 independent reflections
Radiation source: fine-focus sealed tube	10611 reflections with *I* > 2σ(*I*)
graphite	*R*_int_ = 0.073
phi and ω scans	θ_max_ = 25.0°, θ_min_ = 1.2°
Absorption correction: multi-scan (*SADABS*; Bruker, 2007)	*h* = −35→41
*T*_min_ = 0.769, *T*_max_ = 0.841	*k* = −11→11
44864 measured reflections	*l* = −32→33

### Refinement

**Table:**

Refinement on *F*^2^	Secondary atom site location: difference Fourier map
Least-squares matrix: full	Hydrogen site location: inferred from neighbouring sites
*R*[*F*^2^ > 2σ(*F*^2^)] = 0.073	H-atom parameters constrained
*wR*(*F*^2^) = 0.206	*w* = 1/[σ^2^(*F*_o_^2^) + (0.1044*P*)^2^ + 37.5508*P*] where *P* = (*F*_o_^2^ + 2*F*_c_^2^)/3
*S* = 1.02	(Δ/σ)_max_ = 0.018
15556 reflections	Δρ_max_ = 5.46 e Å^−^^3^
999 parameters	Δρ_min_ = −1.00 e Å^−^^3^
1092 restraints	Extinction correction: *SHELXL97* (Sheldrick, 2008)
Primary atom site location: structure-invariant direct methods	

### Fractional atomic coordinates and isotropic or equivalent isotropic displacement parameters (Å^2^)

**Table:**

	*x*	*y*	*z*	*U*_iso_*/*U*_eq_	
Pr1	0.219711 (13)	0.29580 (4)	0.368366 (17)	0.02660 (15)	
Pr2	0.278908 (14)	0.67515 (5)	0.394883 (17)	0.02809 (15)	
O1	0.17085 (18)	0.3021 (7)	0.2804 (2)	0.0437 (16)	
O2	0.1138 (2)	0.1885 (8)	0.2729 (3)	0.059 (2)	
H2	0.1245	0.1703	0.3025	0.088*	
O3	0.15337 (18)	0.1823 (6)	0.3664 (2)	0.0421 (15)	
O4	0.20493 (17)	0.0867 (6)	0.4208 (2)	0.0359 (14)	
O5	0.28415 (17)	0.1920 (6)	0.4161 (2)	0.0384 (15)	
O6	0.29358 (18)	0.4233 (6)	0.4144 (2)	0.0403 (15)	
O7	0.21606 (19)	0.4056 (6)	0.4453 (2)	0.0423 (15)	
O8	0.24253 (19)	0.6234 (6)	0.4517 (2)	0.0417 (15)	
O9	0.20635 (17)	0.5459 (6)	0.3495 (2)	0.0342 (14)	
O10	0.21380 (17)	0.7776 (6)	0.3461 (2)	0.0381 (14)	
O11	0.25600 (18)	0.3470 (6)	0.3101 (2)	0.0384 (15)	
O12	0.2818 (2)	0.5676 (7)	0.3191 (2)	0.0494 (17)	
O13	0.29395 (17)	0.8839 (6)	0.3427 (2)	0.0375 (14)	
O14	0.34550 (18)	0.7831 (7)	0.3943 (2)	0.0468 (17)	
O15	0.3288 (2)	0.6725 (7)	0.4825 (2)	0.0494 (17)	
O16	0.3870 (2)	0.7699 (10)	0.4869 (3)	0.069 (2)	
H16	0.3853	0.7336	0.4597	0.104*	
O1W	0.22485 (18)	0.0606 (6)	0.3258 (2)	0.0383 (15)	
H1WB	0.2196	−0.0245	0.3220	0.057*	
H1WA	0.2449	0.0833	0.3180	0.057*	
O2W	0.27289 (18)	0.9115 (6)	0.4382 (2)	0.0403 (15)	
H2WB	0.2493	0.9065	0.4366	0.060*	
H2WA	0.2800	0.9923	0.4337	0.060*	
C1	0.1391 (3)	0.2616 (10)	0.2546 (4)	0.043 (2)	
C2	0.1242 (3)	0.2919 (11)	0.2005 (4)	0.0470 (19)	
C3	0.0891 (3)	0.2376 (13)	0.1702 (4)	0.060 (2)	
H3	0.0733	0.1802	0.1836	0.072*	
C4	0.0773 (3)	0.2685 (13)	0.1191 (4)	0.062 (2)	
H4	0.0535	0.2308	0.0989	0.074*	
C5	0.0999 (4)	0.3538 (13)	0.0978 (4)	0.063 (2)	
C6	0.1349 (3)	0.4046 (12)	0.1290 (4)	0.061 (2)	
H6	0.1510	0.4607	0.1156	0.073*	
C7	0.1469 (3)	0.3760 (12)	0.1792 (4)	0.056 (2)	
H7	0.1708	0.4136	0.1991	0.067*	
C8	0.0869 (4)	0.3882 (14)	0.0420 (4)	0.074 (3)	
C9	0.0480 (6)	0.3159 (19)	0.0139 (6)	0.122 (5)	
H9A	0.0515	0.2144	0.0155	0.183*	
H9B	0.0402	0.3459	−0.0205	0.183*	
H9C	0.0277	0.3418	0.0289	0.183*	
C10	0.1168 (5)	0.3347 (18)	0.0178 (5)	0.106 (4)	
H10A	0.1404	0.3921	0.0282	0.159*	
H10B	0.1059	0.3401	−0.0179	0.159*	
H10C	0.1234	0.2375	0.0275	0.159*	
C11	0.0783 (6)	0.5450 (18)	0.0339 (6)	0.126 (5)	
H11A	0.0575	0.5712	0.0481	0.190*	
H11B	0.0700	0.5651	−0.0013	0.190*	
H11C	0.1018	0.5986	0.0498	0.190*	
C12	0.1686 (3)	0.0853 (9)	0.3980 (3)	0.0351 (18)	
C13	0.1426 (3)	−0.0332 (10)	0.4055 (4)	0.0421 (18)	
C14	0.1035 (3)	−0.0393 (11)	0.3808 (4)	0.050 (2)	
H14	0.0919	0.0330	0.3586	0.061*	
C15	0.0802 (3)	−0.1527 (11)	0.3882 (5)	0.059 (2)	
H15	0.0531	−0.1532	0.3714	0.071*	
C16	0.0961 (3)	−0.2624 (11)	0.4195 (5)	0.059 (2)	
C17	0.1353 (3)	−0.2543 (11)	0.4440 (4)	0.058 (2)	
H17	0.1469	−0.3271	0.4660	0.070*	
C18	0.1589 (3)	−0.1440 (10)	0.4377 (4)	0.0496 (19)	
H18	0.1858	−0.1434	0.4552	0.059*	
C19	0.0713 (4)	−0.3889 (13)	0.4291 (6)	0.074 (3)	
C20	0.0774 (5)	−0.4049 (16)	0.4861 (6)	0.104 (4)	
H20A	0.0606	−0.3385	0.4964	0.156*	
H20B	0.1047	−0.3857	0.5040	0.156*	
H20C	0.0708	−0.5002	0.4932	0.156*	
C21	0.0857 (4)	−0.5243 (13)	0.4113 (6)	0.082 (3)	
H21A	0.0711	−0.6037	0.4185	0.123*	
H21B	0.1135	−0.5367	0.4281	0.123*	
H21C	0.0816	−0.5188	0.3758	0.123*	
C22	0.0286 (4)	−0.3700 (16)	0.4035 (7)	0.110 (4)	
H22A	0.0246	−0.3565	0.3683	0.165*	
H22B	0.0189	−0.2881	0.4169	0.165*	
H22C	0.0143	−0.4531	0.4085	0.165*	
C23	0.3064 (3)	0.2987 (9)	0.4273 (3)	0.0357 (18)	
C24	0.3482 (3)	0.2792 (11)	0.4589 (4)	0.0464 (19)	
C25	0.3606 (3)	0.1495 (11)	0.4818 (4)	0.055 (2)	
H25	0.3430	0.0733	0.4757	0.066*	
C26	0.3988 (3)	0.1308 (13)	0.5135 (5)	0.069 (2)	
H26	0.4062	0.0421	0.5281	0.083*	
C27	0.4254 (4)	0.2384 (15)	0.5237 (6)	0.087 (3)	
C28	0.4133 (4)	0.3679 (14)	0.4968 (5)	0.081 (3)	
H28	0.4316	0.4417	0.5008	0.097*	
C29	0.3758 (3)	0.3872 (12)	0.4654 (5)	0.066 (2)	
H29	0.3689	0.4730	0.4484	0.079*	
C30	0.4666 (5)	0.221 (2)	0.5597 (8)	0.131 (5)	
C31	0.4754 (6)	0.073 (2)	0.5814 (9)	0.184 (8)	
H31A	0.4749	0.0068	0.5551	0.276*	
H31B	0.4557	0.0467	0.5973	0.276*	
H31C	0.5012	0.0722	0.6056	0.276*	
C32	0.4994 (6)	0.277 (3)	0.5377 (9)	0.182 (7)	
H32A	0.5051	0.2066	0.5159	0.273*	
H32B	0.5230	0.2971	0.5644	0.273*	
H32C	0.4903	0.3629	0.5191	0.273*	
C33	0.4684 (6)	0.311 (2)	0.6046 (8)	0.174 (6)	
H33A	0.4911	0.2836	0.6318	0.260*	
H33B	0.4447	0.2973	0.6143	0.260*	
H33C	0.4707	0.4093	0.5966	0.260*	
C34	0.2270 (3)	0.5196 (9)	0.4683 (3)	0.0361 (19)	
C35	0.2207 (3)	0.5395 (9)	0.5185 (3)	0.0392 (17)	
C36	0.1985 (3)	0.4442 (10)	0.5365 (3)	0.0442 (19)	
H36	0.1873	0.3650	0.5177	0.053*	
C37	0.1929 (3)	0.4672 (10)	0.5829 (4)	0.050 (2)	
H37	0.1773	0.4034	0.5943	0.060*	
C38	0.2097 (3)	0.5814 (11)	0.6131 (4)	0.0542 (19)	
C39	0.2324 (3)	0.6772 (11)	0.5932 (4)	0.053 (2)	
H39	0.2440	0.7564	0.6118	0.064*	
C40	0.2375 (3)	0.6553 (10)	0.5474 (4)	0.0454 (18)	
H40	0.2525	0.7197	0.5353	0.055*	
C41	0.2040 (4)	0.6068 (13)	0.6639 (5)	0.075 (3)	
C42	0.1835 (5)	0.4932 (14)	0.6816 (5)	0.089 (4)	
H42A	0.1842	0.5117	0.7157	0.134*	
H42B	0.1964	0.4040	0.6798	0.134*	
H42C	0.1564	0.4888	0.6611	0.134*	
C43	0.2483 (5)	0.5993 (17)	0.7042 (5)	0.106 (4)	
H43A	0.2466	0.6221	0.7370	0.160*	
H43B	0.2655	0.6664	0.6949	0.160*	
H43C	0.2589	0.5049	0.7044	0.160*	
C44	0.1931 (5)	0.7564 (15)	0.6704 (5)	0.091 (4)	
H44A	0.1665	0.7744	0.6497	0.136*	
H44B	0.2112	0.8194	0.6609	0.136*	
H44C	0.1946	0.7726	0.7048	0.136*	
C45	0.1919 (2)	0.6687 (8)	0.3352 (3)	0.0300 (17)	
C46	0.1501 (3)	0.6823 (9)	0.3038 (3)	0.0374 (17)	
C47	0.1234 (3)	0.5714 (10)	0.3011 (4)	0.0440 (19)	
H47	0.1312	0.4909	0.3210	0.053*	
C48	0.0850 (3)	0.5809 (11)	0.2685 (4)	0.056 (2)	
H48	0.0674	0.5057	0.2667	0.067*	
C49	0.0726 (3)	0.6994 (12)	0.2390 (5)	0.067 (2)	
C50	0.0997 (3)	0.8112 (11)	0.2450 (4)	0.056 (2)	
H50	0.0918	0.8940	0.2264	0.068*	
C51	0.1369 (3)	0.8038 (10)	0.2767 (4)	0.0462 (19)	
H51	0.1538	0.8819	0.2803	0.055*	
C52	0.0328 (4)	0.7049 (17)	0.1989 (7)	0.098 (4)	
C53	0.0203 (5)	0.571 (2)	0.1728 (7)	0.146 (5)	
H53A	0.0011	0.5900	0.1411	0.219*	
H53B	0.0086	0.5115	0.1925	0.219*	
H53C	0.0429	0.5242	0.1675	0.219*	
C54	0.0009 (6)	0.724 (2)	0.2283 (8)	0.159 (6)	
H54A	0.0116	0.6895	0.2618	0.239*	
H54B	−0.0227	0.6720	0.2117	0.239*	
H54C	−0.0055	0.8236	0.2292	0.239*	
C55	0.0282 (6)	0.810 (2)	0.1620 (8)	0.166 (6)	
H55A	0.0073	0.7824	0.1328	0.249*	
H55B	0.0526	0.8199	0.1535	0.249*	
H55C	0.0215	0.8988	0.1744	0.249*	
C56	0.2719 (3)	0.4525 (9)	0.2959 (3)	0.0339 (19)	
C57	0.2782 (3)	0.4395 (9)	0.2452 (3)	0.0427 (18)	
C58	0.2626 (3)	0.3265 (10)	0.2135 (4)	0.054 (2)	
H58	0.2476	0.2581	0.2239	0.065*	
C59	0.2685 (4)	0.3127 (11)	0.1679 (4)	0.059 (2)	
H59	0.2574	0.2354	0.1478	0.071*	
C60	0.2909 (4)	0.4128 (11)	0.1505 (4)	0.060 (2)	
C61	0.3061 (3)	0.5266 (11)	0.1818 (4)	0.055 (2)	
H61	0.3206	0.5964	0.1711	0.066*	
C62	0.3005 (3)	0.5396 (10)	0.2288 (4)	0.049 (2)	
H62	0.3116	0.6159	0.2493	0.059*	
C63	0.2979 (5)	0.3977 (14)	0.0998 (5)	0.086 (3)	
C64	0.3155 (5)	0.5161 (16)	0.0826 (5)	0.109 (4)	
H64A	0.3417	0.5319	0.1048	0.164*	
H64B	0.2996	0.5995	0.0818	0.164*	
H64C	0.3172	0.4968	0.0496	0.164*	
C65	0.3074 (5)	0.2502 (16)	0.0890 (6)	0.103 (4)	
H65A	0.3093	0.2448	0.0554	0.155*	
H65B	0.2868	0.1874	0.0923	0.155*	
H65C	0.3323	0.2224	0.1121	0.155*	
C66	0.2514 (6)	0.4129 (18)	0.0581 (6)	0.125 (5)	
H66A	0.2411	0.5065	0.0605	0.188*	
H66B	0.2341	0.3431	0.0658	0.188*	
H66C	0.2528	0.3976	0.0248	0.188*	
C67	0.3302 (3)	0.8839 (10)	0.3644 (3)	0.0388 (19)	
C68	0.3551 (3)	1.0026 (10)	0.3575 (4)	0.0465 (19)	
C69	0.3387 (3)	1.1192 (11)	0.3268 (5)	0.060 (2)	
H69	0.3115	1.1199	0.3105	0.072*	
C70	0.3617 (3)	1.2337 (12)	0.3200 (5)	0.069 (2)	
H70	0.3499	1.3074	0.2984	0.082*	
C71	0.4014 (3)	1.2384 (12)	0.3448 (5)	0.069 (2)	
C72	0.4180 (3)	1.1232 (12)	0.3761 (5)	0.066 (2)	
H72	0.4449	1.1234	0.3933	0.080*	
C73	0.3948 (3)	1.0103 (12)	0.3817 (4)	0.058 (2)	
H73	0.4066	0.9360	0.4028	0.070*	
C74	0.4260 (4)	1.3648 (14)	0.3378 (7)	0.089 (3)	
C75	0.4082 (4)	1.5011 (14)	0.3530 (7)	0.105 (4)	
H75A	0.4122	1.5007	0.3884	0.158*	
H75B	0.3802	1.5047	0.3358	0.158*	
H75C	0.4210	1.5829	0.3440	0.158*	
C76	0.4206 (5)	1.3788 (18)	0.2805 (7)	0.120 (4)	
H76A	0.4332	1.4647	0.2743	0.180*	
H76B	0.3928	1.3820	0.2627	0.180*	
H76C	0.4325	1.2982	0.2694	0.180*	
C77	0.4685 (5)	1.3515 (18)	0.3629 (8)	0.133 (5)	
H77A	0.4729	1.3430	0.3984	0.199*	
H77B	0.4820	1.4344	0.3561	0.199*	
H77C	0.4786	1.2682	0.3508	0.199*	
C78	0.3618 (3)	0.7088 (12)	0.5065 (4)	0.053 (2)	
C79	0.3773 (4)	0.6840 (13)	0.5612 (4)	0.062 (2)	
C80	0.4139 (4)	0.7294 (15)	0.5894 (4)	0.074 (3)	
H80	0.4305	0.7753	0.5739	0.089*	
C81	0.4269 (4)	0.7086 (15)	0.6408 (5)	0.079 (3)	
H81	0.4519	0.7399	0.6594	0.095*	
C82	0.4022 (4)	0.6406 (16)	0.6645 (5)	0.084 (3)	
C83	0.3659 (4)	0.5960 (14)	0.6357 (4)	0.073 (3)	
H83	0.3494	0.5488	0.6512	0.087*	
C84	0.3525 (4)	0.6175 (14)	0.5850 (4)	0.069 (2)	
H84	0.3272	0.5880	0.5668	0.083*	
C85	0.4149 (6)	0.616 (2)	0.7213 (5)	0.110 (4)	
C86	0.3854 (6)	0.662 (2)	0.7440 (7)	0.153 (5)	
H86A	0.3965	0.6622	0.7797	0.230*	
H86B	0.3631	0.5984	0.7347	0.230*	
H86C	0.3768	0.7567	0.7328	0.230*	
C87	0.4299 (7)	0.472 (2)	0.7335 (7)	0.170 (6)	
H87A	0.4584	0.4731	0.7423	0.255*	
H87B	0.4195	0.4117	0.7050	0.255*	
H87C	0.4217	0.4373	0.7612	0.255*	
C88	0.4479 (7)	0.712 (2)	0.7468 (7)	0.164 (6)	
H88A	0.4427	0.8058	0.7328	0.245*	
H88B	0.4722	0.6767	0.7423	0.245*	
H88C	0.4502	0.7153	0.7819	0.245*	

### Atomic displacement parameters (Å^2^)

**Table:**

	*U*^11^	*U*^22^	*U*^33^	*U*^12^	*U*^13^	*U*^23^
Pr1	0.0312 (3)	0.0197 (2)	0.0273 (3)	−0.00240 (18)	0.00606 (19)	0.00266 (17)
Pr2	0.0339 (3)	0.0216 (2)	0.0278 (3)	−0.00361 (18)	0.0075 (2)	0.00178 (18)
O1	0.039 (4)	0.049 (4)	0.037 (4)	−0.001 (3)	0.001 (3)	0.011 (3)
O2	0.049 (4)	0.068 (5)	0.045 (4)	−0.018 (4)	−0.008 (3)	0.014 (4)
O3	0.038 (4)	0.044 (4)	0.040 (4)	−0.007 (3)	0.005 (3)	0.011 (3)
O4	0.035 (4)	0.034 (3)	0.038 (3)	−0.003 (3)	0.008 (3)	0.012 (3)
O5	0.036 (3)	0.025 (3)	0.048 (4)	−0.005 (3)	0.003 (3)	0.007 (3)
O6	0.044 (4)	0.030 (3)	0.043 (4)	0.006 (3)	0.008 (3)	0.005 (3)
O7	0.061 (4)	0.033 (3)	0.037 (4)	−0.011 (3)	0.021 (3)	−0.005 (3)
O8	0.055 (4)	0.035 (3)	0.040 (4)	−0.011 (3)	0.021 (3)	0.000 (3)
O9	0.043 (3)	0.023 (3)	0.030 (3)	0.006 (3)	0.000 (3)	0.006 (2)
O10	0.033 (3)	0.027 (3)	0.046 (4)	−0.005 (3)	−0.001 (3)	0.006 (3)
O11	0.054 (4)	0.027 (3)	0.042 (4)	−0.005 (3)	0.026 (3)	0.001 (3)
O12	0.074 (5)	0.039 (4)	0.041 (4)	−0.010 (3)	0.027 (4)	−0.008 (3)
O13	0.032 (4)	0.033 (3)	0.047 (4)	−0.004 (3)	0.010 (3)	0.002 (3)
O14	0.037 (4)	0.050 (4)	0.050 (4)	−0.007 (3)	0.008 (3)	0.023 (3)
O15	0.053 (4)	0.050 (4)	0.039 (4)	−0.003 (3)	0.003 (3)	0.004 (3)
O16	0.058 (5)	0.092 (6)	0.050 (5)	−0.016 (4)	0.003 (4)	0.019 (4)
O1W	0.063 (4)	0.019 (3)	0.034 (3)	−0.004 (3)	0.017 (3)	−0.005 (2)
O2W	0.052 (4)	0.020 (3)	0.047 (4)	−0.002 (3)	0.010 (3)	−0.008 (3)
C1	0.049 (5)	0.033 (4)	0.043 (5)	−0.004 (4)	0.007 (4)	0.004 (4)
C2	0.047 (4)	0.048 (4)	0.041 (4)	−0.003 (3)	0.006 (3)	0.008 (3)
C3	0.056 (4)	0.066 (5)	0.049 (4)	−0.003 (4)	0.001 (4)	0.006 (4)
C4	0.059 (5)	0.068 (5)	0.049 (4)	0.001 (4)	0.002 (4)	0.005 (4)
C5	0.070 (4)	0.066 (4)	0.046 (4)	0.009 (4)	0.006 (4)	0.006 (4)
C6	0.065 (5)	0.062 (5)	0.050 (4)	−0.007 (4)	0.007 (4)	0.008 (4)
C7	0.057 (4)	0.057 (4)	0.048 (4)	−0.003 (4)	0.007 (4)	0.008 (4)
C8	0.096 (7)	0.079 (6)	0.039 (5)	0.027 (6)	0.008 (5)	0.011 (5)
C9	0.145 (10)	0.135 (10)	0.064 (8)	0.019 (9)	−0.003 (8)	0.020 (8)
C10	0.122 (9)	0.130 (10)	0.062 (7)	0.023 (8)	0.021 (7)	0.021 (7)
C11	0.176 (11)	0.110 (9)	0.072 (8)	0.031 (9)	0.003 (8)	0.004 (8)
C12	0.035 (4)	0.037 (4)	0.036 (4)	−0.001 (3)	0.015 (3)	0.008 (3)
C13	0.036 (4)	0.040 (4)	0.052 (4)	−0.004 (3)	0.016 (3)	0.006 (3)
C14	0.038 (4)	0.046 (4)	0.063 (4)	−0.004 (3)	0.008 (3)	0.014 (4)
C15	0.044 (4)	0.054 (4)	0.076 (5)	−0.013 (4)	0.010 (4)	0.008 (4)
C16	0.045 (4)	0.043 (4)	0.089 (5)	−0.013 (3)	0.022 (4)	0.008 (4)
C17	0.047 (4)	0.043 (4)	0.081 (5)	−0.007 (4)	0.014 (4)	0.018 (4)
C18	0.038 (4)	0.041 (4)	0.068 (4)	−0.004 (3)	0.014 (3)	0.015 (4)
C19	0.056 (5)	0.050 (5)	0.123 (7)	−0.016 (5)	0.038 (5)	0.002 (6)
C20	0.108 (8)	0.076 (8)	0.141 (10)	−0.031 (7)	0.057 (8)	0.003 (8)
C21	0.084 (7)	0.052 (6)	0.115 (8)	−0.020 (6)	0.038 (6)	−0.001 (6)
C22	0.082 (7)	0.075 (7)	0.168 (10)	−0.030 (6)	0.032 (7)	0.012 (8)
C23	0.032 (4)	0.035 (4)	0.043 (4)	0.003 (3)	0.015 (4)	0.007 (4)
C24	0.035 (4)	0.042 (4)	0.058 (4)	0.002 (3)	0.008 (3)	−0.001 (4)
C25	0.038 (4)	0.053 (4)	0.066 (4)	−0.001 (3)	0.002 (4)	0.007 (4)
C26	0.046 (4)	0.065 (5)	0.081 (5)	0.006 (4)	−0.006 (4)	0.009 (4)
C27	0.055 (4)	0.073 (5)	0.105 (5)	0.005 (4)	−0.018 (4)	−0.003 (5)
C28	0.052 (5)	0.068 (5)	0.101 (5)	−0.004 (4)	−0.012 (4)	−0.002 (5)
C29	0.040 (4)	0.054 (4)	0.088 (5)	0.000 (4)	−0.004 (4)	0.001 (4)
C30	0.079 (7)	0.108 (8)	0.160 (9)	0.002 (7)	−0.034 (7)	−0.009 (8)
C31	0.117 (11)	0.141 (13)	0.218 (14)	0.019 (11)	−0.069 (11)	0.005 (12)
C32	0.104 (12)	0.189 (14)	0.210 (14)	0.008 (12)	−0.019 (12)	0.002 (13)
C33	0.117 (10)	0.158 (11)	0.186 (11)	0.003 (9)	−0.049 (9)	−0.007 (10)
C34	0.049 (5)	0.029 (4)	0.030 (4)	−0.001 (4)	0.010 (4)	0.004 (3)
C35	0.056 (4)	0.031 (3)	0.034 (4)	−0.006 (3)	0.018 (3)	0.001 (3)
C36	0.060 (4)	0.036 (4)	0.041 (4)	−0.010 (3)	0.022 (4)	−0.004 (3)
C37	0.071 (5)	0.042 (4)	0.043 (4)	−0.008 (4)	0.028 (4)	−0.002 (3)
C38	0.085 (4)	0.041 (4)	0.044 (4)	−0.006 (4)	0.030 (4)	−0.003 (3)
C39	0.077 (5)	0.045 (4)	0.042 (4)	−0.011 (4)	0.022 (4)	−0.002 (3)
C40	0.064 (4)	0.036 (4)	0.039 (4)	−0.009 (3)	0.020 (3)	−0.001 (3)
C41	0.129 (7)	0.054 (5)	0.055 (5)	−0.011 (6)	0.048 (5)	−0.006 (5)
C42	0.147 (9)	0.076 (7)	0.066 (7)	−0.009 (7)	0.063 (7)	−0.004 (6)
C43	0.161 (10)	0.087 (8)	0.069 (8)	−0.012 (8)	0.032 (8)	−0.004 (7)
C44	0.145 (9)	0.072 (7)	0.074 (7)	0.011 (7)	0.061 (7)	−0.004 (6)
C45	0.032 (4)	0.028 (4)	0.029 (4)	0.003 (3)	0.007 (3)	0.007 (3)
C46	0.035 (4)	0.032 (3)	0.040 (4)	0.004 (3)	0.003 (3)	0.002 (3)
C47	0.039 (4)	0.032 (4)	0.055 (4)	−0.004 (3)	0.005 (3)	0.003 (3)
C48	0.043 (4)	0.041 (4)	0.073 (5)	−0.006 (4)	0.003 (4)	0.000 (4)
C49	0.045 (4)	0.058 (4)	0.082 (5)	0.002 (4)	−0.007 (4)	0.000 (4)
C50	0.048 (4)	0.042 (4)	0.066 (5)	0.003 (4)	−0.003 (4)	0.009 (4)
C51	0.043 (4)	0.034 (4)	0.052 (4)	−0.003 (3)	−0.001 (3)	0.009 (3)
C52	0.050 (6)	0.090 (7)	0.123 (8)	0.012 (6)	−0.022 (6)	0.002 (6)
C53	0.100 (9)	0.129 (10)	0.153 (10)	0.000 (8)	−0.051 (8)	−0.003 (9)
C54	0.099 (9)	0.165 (10)	0.169 (11)	0.021 (9)	−0.031 (9)	−0.019 (9)
C55	0.110 (9)	0.150 (10)	0.175 (11)	−0.018 (9)	−0.058 (9)	0.023 (9)
C56	0.049 (5)	0.030 (4)	0.030 (4)	0.000 (4)	0.023 (4)	0.004 (4)
C57	0.066 (4)	0.032 (3)	0.037 (4)	−0.006 (3)	0.027 (3)	0.001 (3)
C58	0.082 (5)	0.042 (4)	0.045 (4)	−0.015 (4)	0.029 (4)	−0.005 (3)
C59	0.092 (5)	0.045 (4)	0.045 (4)	−0.014 (4)	0.030 (4)	−0.002 (4)
C60	0.099 (5)	0.045 (4)	0.048 (4)	−0.010 (4)	0.038 (4)	−0.002 (3)
C61	0.084 (5)	0.044 (4)	0.047 (4)	−0.011 (4)	0.034 (4)	0.004 (4)
C62	0.079 (5)	0.035 (4)	0.042 (4)	−0.009 (4)	0.031 (4)	0.000 (3)
C63	0.150 (8)	0.056 (6)	0.068 (6)	−0.009 (6)	0.056 (6)	−0.002 (5)
C64	0.186 (12)	0.091 (9)	0.077 (8)	−0.013 (9)	0.080 (8)	0.011 (7)
C65	0.172 (10)	0.071 (7)	0.087 (8)	0.005 (8)	0.068 (7)	−0.003 (7)
C66	0.199 (12)	0.095 (9)	0.080 (8)	−0.011 (9)	0.039 (9)	0.007 (8)
C67	0.044 (5)	0.036 (4)	0.040 (4)	−0.002 (4)	0.018 (4)	0.002 (4)
C68	0.035 (4)	0.041 (4)	0.066 (4)	−0.006 (3)	0.019 (3)	0.008 (4)
C69	0.042 (4)	0.046 (4)	0.091 (5)	−0.004 (4)	0.017 (4)	0.009 (4)
C70	0.049 (4)	0.048 (4)	0.108 (5)	−0.007 (4)	0.021 (4)	0.016 (4)
C71	0.050 (4)	0.049 (4)	0.110 (5)	−0.012 (4)	0.024 (4)	0.008 (4)
C72	0.043 (4)	0.057 (4)	0.096 (5)	−0.013 (4)	0.015 (4)	0.008 (4)
C73	0.044 (4)	0.051 (4)	0.078 (5)	−0.006 (3)	0.015 (4)	0.010 (4)
C74	0.064 (5)	0.059 (5)	0.141 (7)	−0.009 (5)	0.027 (5)	0.010 (6)
C75	0.091 (8)	0.062 (7)	0.168 (10)	−0.016 (6)	0.045 (7)	0.009 (7)
C76	0.099 (8)	0.088 (8)	0.180 (10)	−0.024 (7)	0.052 (8)	0.018 (8)
C77	0.084 (8)	0.096 (9)	0.214 (12)	−0.025 (8)	0.036 (9)	0.012 (9)
C78	0.053 (5)	0.056 (5)	0.045 (5)	−0.003 (4)	0.008 (4)	0.006 (4)
C79	0.063 (5)	0.074 (5)	0.041 (4)	−0.001 (4)	0.003 (4)	0.006 (4)
C80	0.072 (5)	0.090 (5)	0.050 (4)	−0.005 (5)	0.002 (4)	0.011 (4)
C81	0.079 (5)	0.096 (5)	0.050 (5)	−0.001 (5)	−0.002 (4)	0.007 (5)
C82	0.096 (5)	0.100 (5)	0.048 (4)	0.012 (5)	0.006 (4)	0.009 (4)
C83	0.079 (5)	0.086 (5)	0.046 (4)	−0.002 (5)	0.009 (4)	0.013 (4)
C84	0.073 (5)	0.078 (5)	0.050 (4)	−0.003 (4)	0.006 (4)	0.010 (4)
C85	0.132 (8)	0.125 (8)	0.053 (6)	0.036 (7)	−0.002 (6)	0.000 (6)
C86	0.172 (11)	0.188 (11)	0.082 (9)	0.027 (10)	0.011 (9)	0.008 (9)
C87	0.212 (11)	0.171 (11)	0.088 (9)	0.043 (10)	−0.016 (9)	0.006 (9)
C88	0.188 (11)	0.180 (11)	0.089 (9)	0.006 (10)	−0.012 (9)	−0.006 (9)

### Geometric parameters (Å, °)

**Table:**

Pr1—O11	2.395 (6)	C36—H36	0.9300
Pr1—O9	2.422 (5)	C37—C38	1.383 (14)
Pr1—O7	2.423 (6)	C37—H37	0.9300
Pr1—O5	2.469 (6)	C38—C39	1.421 (14)
Pr1—O1W	2.539 (5)	C38—C41	1.511 (14)
Pr1—O3	2.552 (6)	C39—C40	1.358 (13)
Pr1—O1	2.555 (6)	C39—H39	0.9300
Pr1—O4	2.593 (5)	C40—H40	0.9300
Pr1—O6	2.807 (6)	C41—C42	1.453 (16)
Pr2—O8	2.366 (6)	C41—C44	1.482 (17)
Pr2—O12	2.375 (6)	C41—C43	1.64 (2)
Pr2—O6	2.447 (6)	C42—H42A	0.9600
Pr2—O10	2.490 (6)	C42—H42B	0.9600
Pr2—O14	2.557 (6)	C42—H42C	0.9600
Pr2—O15	2.562 (7)	C43—H43A	0.9600
Pr2—O2W	2.567 (5)	C43—H43B	0.9600
Pr2—O13	2.589 (6)	C43—H43C	0.9600
Pr2—O9	2.775 (6)	C44—H44A	0.9600
O1—C1	1.199 (11)	C44—H44B	0.9600
O2—C1	1.340 (11)	C44—H44C	0.9600
O2—H2	0.8200	C45—C46	1.481 (12)
O3—C12	1.273 (10)	C46—C51	1.371 (12)
O4—C12	1.251 (10)	C46—C47	1.389 (12)
O5—C23	1.253 (10)	C47—C48	1.394 (13)
O6—C23	1.267 (10)	C47—H47	0.9300
O7—C34	1.249 (10)	C48—C49	1.377 (15)
O8—C34	1.270 (10)	C48—H48	0.9300
O9—C45	1.276 (9)	C49—C50	1.395 (15)
O10—C45	1.262 (10)	C49—C52	1.516 (16)
O11—C56	1.258 (10)	C50—C51	1.352 (13)
O12—C56	1.256 (10)	C50—H50	0.9300
O13—C67	1.243 (10)	C51—H51	0.9300
O14—C67	1.273 (11)	C52—C55	1.40 (2)
O15—C78	1.207 (12)	C52—C53	1.45 (2)
O16—C78	1.303 (12)	C52—C54	1.58 (3)
O16—H16	0.8200	C53—H53A	0.9600
O1W—H1WB	0.8202	C53—H53B	0.9600
O1W—H1WA	0.8244	C53—H53C	0.9600
O2W—H2WB	0.8200	C54—H54A	0.9600
O2W—H2WA	0.8200	C54—H54B	0.9600
C1—C2	1.474 (13)	C54—H54C	0.9600
C2—C3	1.374 (14)	C55—H55A	0.9600
C2—C7	1.379 (14)	C55—H55B	0.9600
C3—C4	1.395 (15)	C55—H55C	0.9600
C3—H3	0.9300	C56—C57	1.502 (11)
C4—C5	1.382 (16)	C57—C62	1.384 (12)
C4—H4	0.9300	C57—C58	1.388 (13)
C5—C6	1.368 (15)	C58—C59	1.357 (14)
C5—C8	1.524 (15)	C58—H58	0.9300
C6—C7	1.369 (14)	C59—C60	1.405 (14)
C6—H6	0.9300	C59—H59	0.9300
C7—H7	0.9300	C60—C61	1.386 (14)
C8—C10	1.496 (19)	C60—C63	1.513 (15)
C8—C11	1.51 (2)	C61—C62	1.391 (13)
C8—C9	1.53 (2)	C61—H61	0.9300
C9—H9A	0.9600	C62—H62	0.9300
C9—H9B	0.9600	C63—C64	1.423 (18)
C9—H9C	0.9600	C63—C65	1.477 (19)
C10—H10A	0.9600	C63—C66	1.71 (2)
C10—H10B	0.9600	C64—H64A	0.9600
C10—H10C	0.9600	C64—H64B	0.9600
C11—H11A	0.9600	C64—H64C	0.9600
C11—H11B	0.9600	C65—H65A	0.9600
C11—H11C	0.9600	C65—H65B	0.9600
C12—C13	1.494 (12)	C65—H65C	0.9600
C13—C14	1.346 (13)	C66—H66A	0.9600
C13—C18	1.385 (13)	C66—H66B	0.9600
C14—C15	1.397 (13)	C66—H66C	0.9600
C14—H14	0.9300	C67—C68	1.463 (12)
C15—C16	1.361 (15)	C68—C73	1.364 (13)
C15—H15	0.9300	C68—C69	1.406 (14)
C16—C17	1.353 (14)	C69—C70	1.391 (14)
C16—C19	1.541 (14)	C69—H69	0.9300
C17—C18	1.368 (13)	C70—C71	1.368 (15)
C17—H17	0.9300	C70—H70	0.9300
C18—H18	0.9300	C71—C72	1.406 (17)
C19—C22	1.474 (18)	C71—C74	1.514 (16)
C19—C21	1.505 (17)	C72—C73	1.374 (14)
C19—C20	1.55 (2)	C72—H72	0.9300
C20—H20A	0.9600	C73—H73	0.9300
C20—H20B	0.9600	C74—C77	1.46 (2)
C20—H20C	0.9600	C74—C75	1.537 (19)
C21—H21A	0.9600	C74—C76	1.56 (2)
C21—H21B	0.9600	C75—H75A	0.9600
C21—H21C	0.9600	C75—H75B	0.9600
C22—H22A	0.9600	C75—H75C	0.9600
C22—H22B	0.9600	C76—H76A	0.9600
C22—H22C	0.9600	C76—H76B	0.9600
C23—C24	1.490 (13)	C76—H76C	0.9600
C24—C29	1.378 (14)	C77—H77A	0.9600
C24—C25	1.385 (14)	C77—H77B	0.9600
C25—C26	1.385 (14)	C77—H77C	0.9600
C25—H25	0.9300	C78—C79	1.483 (15)
C26—C27	1.349 (17)	C79—C80	1.367 (16)
C26—H26	0.9300	C79—C84	1.390 (16)
C27—C28	1.428 (18)	C80—C81	1.387 (16)
C27—C30	1.513 (19)	C80—H80	0.9300
C28—C29	1.366 (15)	C81—C82	1.392 (19)
C28—H28	0.9300	C81—H81	0.9300
C29—H29	0.9300	C82—C83	1.359 (18)
C30—C33	1.50 (3)	C82—C85	1.536 (19)
C30—C31	1.51 (3)	C83—C84	1.372 (15)
C30—C32	1.55 (3)	C83—H83	0.9300
C31—H31A	0.9600	C84—H84	0.9300
C31—H31B	0.9600	C85—C86	1.43 (2)
C31—H31C	0.9600	C85—C87	1.45 (2)
C32—H32A	0.9600	C85—C88	1.47 (3)
C32—H32B	0.9600	C86—H86A	0.9600
C32—H32C	0.9600	C86—H86B	0.9600
C33—H33A	0.9600	C86—H86C	0.9600
C33—H33B	0.9600	C87—H87A	0.9600
C33—H33C	0.9600	C87—H87B	0.9600
C34—C35	1.493 (12)	C87—H87C	0.9600
C35—C36	1.378 (12)	C88—H88A	0.9600
C35—C40	1.379 (12)	C88—H88B	0.9600
C36—C37	1.384 (12)	C88—H88C	0.9600
			
O11—Pr1—O9	76.46 (19)	H33A—C33—H33B	109.5
O11—Pr1—O7	134.2 (2)	C30—C33—H33C	109.5
O9—Pr1—O7	73.9 (2)	H33A—C33—H33C	109.5
O11—Pr1—O5	81.8 (2)	H33B—C33—H33C	109.5
O9—Pr1—O5	126.08 (18)	O7—C34—O8	124.7 (8)
O7—Pr1—O5	88.2 (2)	O7—C34—C35	118.9 (8)
O11—Pr1—O1W	74.35 (19)	O8—C34—C35	116.4 (8)
O9—Pr1—O1W	140.98 (18)	C36—C35—C40	119.1 (9)
O7—Pr1—O1W	144.63 (19)	C36—C35—C34	121.2 (8)
O5—Pr1—O1W	74.31 (19)	C40—C35—C34	119.6 (8)
O11—Pr1—O3	137.4 (2)	C35—C36—C37	119.6 (9)
O9—Pr1—O3	106.4 (2)	C35—C36—H36	120.2
O7—Pr1—O3	84.8 (2)	C37—C36—H36	120.2
O5—Pr1—O3	122.45 (19)	C38—C37—C36	122.6 (9)
O1W—Pr1—O3	79.5 (2)	C38—C37—H37	118.7
O11—Pr1—O1	71.5 (2)	C36—C37—H37	118.7
O9—Pr1—O1	74.66 (19)	C37—C38—C39	116.1 (9)
O7—Pr1—O1	130.5 (2)	C37—C38—C41	123.0 (10)
O5—Pr1—O1	141.4 (2)	C39—C38—C41	120.9 (10)
O1W—Pr1—O1	71.90 (19)	C40—C39—C38	121.1 (10)
O3—Pr1—O1	68.7 (2)	C40—C39—H39	119.4
O11—Pr1—O4	140.54 (19)	C38—C39—H39	119.4
O9—Pr1—O4	143.00 (19)	C39—C40—C35	121.4 (9)
O7—Pr1—O4	75.41 (19)	C39—C40—H40	119.3
O5—Pr1—O4	72.59 (18)	C35—C40—H40	119.3
O1W—Pr1—O4	70.14 (19)	C42—C41—C44	118.8 (12)
O3—Pr1—O4	50.37 (18)	C42—C41—C38	114.9 (10)
O1—Pr1—O4	111.84 (19)	C44—C41—C38	112.1 (10)
O11—Pr1—O6	67.6 (2)	C42—C41—C43	101.6 (11)
O9—Pr1—O6	77.36 (18)	C44—C41—C43	100.6 (11)
O7—Pr1—O6	72.25 (19)	C38—C41—C43	106.2 (11)
O5—Pr1—O6	48.72 (17)	C41—C42—H42A	109.5
O1W—Pr1—O6	113.67 (19)	C41—C42—H42B	109.5
O3—Pr1—O6	154.96 (19)	H42A—C42—H42B	109.5
O1—Pr1—O6	134.61 (19)	C41—C42—H42C	109.5
O4—Pr1—O6	112.09 (18)	H42A—C42—H42C	109.5
O8—Pr2—O12	133.2 (2)	H42B—C42—H42C	109.5
O8—Pr2—O6	76.8 (2)	C41—C43—H43A	109.5
O12—Pr2—O6	74.1 (2)	C41—C43—H43B	109.5
O8—Pr2—O10	81.6 (2)	H43A—C43—H43B	109.5
O12—Pr2—O10	87.1 (2)	C41—C43—H43C	109.5
O6—Pr2—O10	126.53 (19)	H43A—C43—H43C	109.5
O8—Pr2—O14	139.7 (2)	H43B—C43—H43C	109.5
O12—Pr2—O14	83.3 (2)	C41—C44—H44A	109.5
O6—Pr2—O14	104.5 (2)	C41—C44—H44B	109.5
O10—Pr2—O14	122.78 (19)	H44A—C44—H44B	109.5
O8—Pr2—O15	73.1 (2)	C41—C44—H44C	109.5
O12—Pr2—O15	130.7 (2)	H44A—C44—H44C	109.5
O6—Pr2—O15	74.7 (2)	H44B—C44—H44C	109.5
O10—Pr2—O15	142.2 (2)	O10—C45—O9	119.8 (7)
O14—Pr2—O15	68.7 (2)	O10—C45—C46	120.2 (7)
O8—Pr2—O2W	74.0 (2)	O9—C45—C46	119.9 (7)
O12—Pr2—O2W	145.0 (2)	C51—C46—C47	118.7 (9)
O6—Pr2—O2W	140.5 (2)	C51—C46—C45	121.0 (8)
O10—Pr2—O2W	74.60 (19)	C47—C46—C45	120.4 (8)
O14—Pr2—O2W	82.1 (2)	C46—C47—C48	119.8 (9)
O15—Pr2—O2W	71.6 (2)	C46—C47—H47	120.1
O8—Pr2—O13	140.8 (2)	C48—C47—H47	120.1
O12—Pr2—O13	75.5 (2)	C49—C48—C47	121.4 (10)
O6—Pr2—O13	142.4 (2)	C49—C48—H48	119.3
O10—Pr2—O13	72.94 (18)	C47—C48—H48	119.3
O14—Pr2—O13	50.02 (19)	C48—C49—C50	116.7 (10)
O15—Pr2—O13	110.5 (2)	C48—C49—C52	122.2 (11)
O2W—Pr2—O13	70.80 (19)	C50—C49—C52	120.9 (12)
O8—Pr2—O9	67.07 (19)	C51—C50—C49	122.5 (10)
O12—Pr2—O9	71.4 (2)	C51—C50—H50	118.8
O6—Pr2—O9	77.60 (18)	C49—C50—H50	118.8
O10—Pr2—O9	48.93 (17)	C50—C51—C46	120.7 (9)
O14—Pr2—O9	153.19 (19)	C50—C51—H51	119.6
O15—Pr2—O9	135.53 (19)	C46—C51—H51	119.6
O2W—Pr2—O9	113.65 (18)	C55—C52—C53	106.5 (17)
O13—Pr2—O9	112.71 (17)	C55—C52—C49	116.8 (14)
C1—O1—Pr1	144.7 (6)	C53—C52—C49	114.8 (12)
C1—O2—H2	109.5	C55—C52—C54	110.7 (16)
C12—O3—Pr1	95.1 (5)	C53—C52—C54	101.8 (15)
C12—O4—Pr1	93.7 (5)	C49—C52—C54	105.1 (14)
C23—O5—Pr1	103.1 (5)	C52—C53—H53A	109.5
C23—O6—Pr2	171.6 (6)	C52—C53—H53B	109.5
C23—O6—Pr1	86.6 (5)	H53A—C53—H53B	109.5
Pr2—O6—Pr1	101.7 (2)	C52—C53—H53C	109.5
C34—O7—Pr1	137.1 (6)	H53A—C53—H53C	109.5
C34—O8—Pr2	141.2 (6)	H53B—C53—H53C	109.5
C45—O9—Pr1	168.0 (5)	C52—C54—H54A	109.5
C45—O9—Pr2	88.7 (5)	C52—C54—H54B	109.5
Pr1—O9—Pr2	103.32 (19)	H54A—C54—H54B	109.5
C45—O10—Pr2	102.6 (5)	C52—C54—H54C	109.5
C56—O11—Pr1	138.4 (5)	H54A—C54—H54C	109.5
C56—O12—Pr2	138.5 (6)	H54B—C54—H54C	109.5
C67—O13—Pr2	94.3 (5)	C52—C55—H55A	109.5
C67—O14—Pr2	95.0 (5)	C52—C55—H55B	109.5
C78—O15—Pr2	143.7 (7)	H55A—C55—H55B	109.5
C78—O16—H16	109.5	C52—C55—H55C	109.5
Pr1—O1W—H1WB	149.7	H55A—C55—H55C	109.5
Pr1—O1W—H1WA	94.8	H55B—C55—H55C	109.5
H1WB—O1W—H1WA	113.3	O12—C56—O11	126.6 (8)
Pr2—O2W—H2WB	98.5	O12—C56—C57	117.5 (7)
Pr2—O2W—H2WA	130.9	O11—C56—C57	115.9 (8)
H2WB—O2W—H2WA	113.3	C62—C57—C58	117.6 (9)
O1—C1—O2	122.6 (9)	C62—C57—C56	120.8 (8)
O1—C1—C2	123.0 (9)	C58—C57—C56	121.5 (8)
O2—C1—C2	114.4 (9)	C59—C58—C57	122.1 (9)
C3—C2—C7	118.4 (10)	C59—C58—H58	119.0
C3—C2—C1	123.2 (9)	C57—C58—H58	119.0
C7—C2—C1	118.4 (9)	C58—C59—C60	121.3 (10)
C2—C3—C4	120.0 (11)	C58—C59—H59	119.3
C2—C3—H3	120.0	C60—C59—H59	119.3
C4—C3—H3	120.0	C61—C60—C59	116.6 (9)
C5—C4—C3	121.6 (11)	C61—C60—C63	121.7 (10)
C5—C4—H4	119.2	C59—C60—C63	121.7 (10)
C3—C4—H4	119.2	C60—C61—C62	121.9 (9)
C6—C5—C4	116.9 (11)	C60—C61—H61	119.0
C6—C5—C8	121.4 (12)	C62—C61—H61	119.0
C4—C5—C8	121.7 (11)	C57—C62—C61	120.4 (9)
C5—C6—C7	122.3 (11)	C57—C62—H62	119.8
C5—C6—H6	118.8	C61—C62—H62	119.8
C7—C6—H6	118.8	C64—C63—C65	121.1 (13)
C6—C7—C2	120.8 (11)	C64—C63—C60	116.7 (11)
C6—C7—H7	119.6	C65—C63—C60	112.8 (11)
C2—C7—H7	119.6	C64—C63—C66	97.5 (12)
C10—C8—C11	113.5 (14)	C65—C63—C66	99.6 (12)
C10—C8—C5	110.7 (11)	C60—C63—C66	104.1 (11)
C11—C8—C5	110.3 (11)	C63—C64—H64A	109.5
C10—C8—C9	105.2 (13)	C63—C64—H64B	109.5
C11—C8—C9	104.2 (12)	H64A—C64—H64B	109.5
C5—C8—C9	112.8 (12)	C63—C64—H64C	109.5
C8—C9—H9A	109.5	H64A—C64—H64C	109.5
C8—C9—H9B	109.5	H64B—C64—H64C	109.5
H9A—C9—H9B	109.5	C63—C65—H65A	109.5
C8—C9—H9C	109.5	C63—C65—H65B	109.5
H9A—C9—H9C	109.5	H65A—C65—H65B	109.5
H9B—C9—H9C	109.5	C63—C65—H65C	109.5
C8—C10—H10A	109.5	H65A—C65—H65C	109.5
C8—C10—H10B	109.5	H65B—C65—H65C	109.5
H10A—C10—H10B	109.5	C63—C66—H66A	109.5
C8—C10—H10C	109.5	C63—C66—H66B	109.5
H10A—C10—H10C	109.5	H66A—C66—H66B	109.5
H10B—C10—H10C	109.5	C63—C66—H66C	109.5
C8—C11—H11A	109.5	H66A—C66—H66C	109.5
C8—C11—H11B	109.5	H66B—C66—H66C	109.5
H11A—C11—H11B	109.5	O13—C67—O14	119.8 (8)
C8—C11—H11C	109.5	O13—C67—C68	120.2 (8)
H11A—C11—H11C	109.5	O14—C67—C68	120.0 (9)
H11B—C11—H11C	109.5	C73—C68—C69	116.0 (9)
O4—C12—O3	120.4 (8)	C73—C68—C67	122.7 (9)
O4—C12—C13	120.9 (8)	C69—C68—C67	121.3 (9)
O3—C12—C13	118.7 (8)	C70—C69—C68	122.4 (10)
C14—C13—C18	117.8 (9)	C70—C69—H69	118.8
C14—C13—C12	122.4 (9)	C68—C69—H69	118.8
C18—C13—C12	119.8 (8)	C71—C70—C69	120.3 (11)
C13—C14—C15	120.9 (10)	C71—C70—H70	119.8
C13—C14—H14	119.6	C69—C70—H70	119.8
C15—C14—H14	119.6	C70—C71—C72	117.7 (10)
C16—C15—C14	121.6 (10)	C70—C71—C74	119.9 (12)
C16—C15—H15	119.2	C72—C71—C74	122.4 (11)
C14—C15—H15	119.2	C73—C72—C71	120.9 (11)
C17—C16—C15	116.6 (10)	C73—C72—H72	119.6
C17—C16—C19	120.2 (11)	C71—C72—H72	119.6
C15—C16—C19	123.2 (10)	C68—C73—C72	122.7 (11)
C16—C17—C18	123.1 (10)	C68—C73—H73	118.6
C16—C17—H17	118.5	C72—C73—H73	118.6
C18—C17—H17	118.5	C77—C74—C71	114.5 (13)
C17—C18—C13	120.0 (10)	C77—C74—C75	112.5 (14)
C17—C18—H18	120.0	C71—C74—C75	108.8 (11)
C13—C18—H18	120.0	C77—C74—C76	107.4 (14)
C22—C19—C21	109.6 (12)	C71—C74—C76	107.2 (12)
C22—C19—C16	111.8 (11)	C75—C74—C76	106.0 (13)
C21—C19—C16	109.3 (10)	C74—C75—H75A	109.5
C22—C19—C20	108.9 (12)	C74—C75—H75B	109.5
C21—C19—C20	107.4 (12)	H75A—C75—H75B	109.5
C16—C19—C20	109.7 (11)	C74—C75—H75C	109.5
C19—C20—H20A	109.5	H75A—C75—H75C	109.5
C19—C20—H20B	109.5	H75B—C75—H75C	109.5
H20A—C20—H20B	109.5	C74—C76—H76A	109.5
C19—C20—H20C	109.5	C74—C76—H76B	109.5
H20A—C20—H20C	109.5	H76A—C76—H76B	109.5
H20B—C20—H20C	109.5	C74—C76—H76C	109.5
C19—C21—H21A	109.5	H76A—C76—H76C	109.5
C19—C21—H21B	109.5	H76B—C76—H76C	109.5
H21A—C21—H21B	109.5	C74—C77—H77A	109.5
C19—C21—H21C	109.5	C74—C77—H77B	109.5
H21A—C21—H21C	109.5	H77A—C77—H77B	109.5
H21B—C21—H21C	109.5	C74—C77—H77C	109.5
C19—C22—H22A	109.5	H77A—C77—H77C	109.5
C19—C22—H22B	109.5	H77B—C77—H77C	109.5
H22A—C22—H22B	109.5	O15—C78—O16	123.6 (10)
C19—C22—H22C	109.5	O15—C78—C79	122.0 (10)
H22A—C22—H22C	109.5	O16—C78—C79	114.4 (10)
H22B—C22—H22C	109.5	C80—C79—C84	118.7 (11)
O5—C23—O6	121.5 (8)	C80—C79—C78	123.0 (11)
O5—C23—C24	119.1 (8)	C84—C79—C78	118.2 (11)
O6—C23—C24	119.3 (8)	C79—C80—C81	121.5 (13)
C29—C24—C25	118.2 (9)	C79—C80—H80	119.3
C29—C24—C23	121.5 (9)	C81—C80—H80	119.3
C25—C24—C23	120.3 (9)	C80—C81—C82	119.7 (13)
C24—C25—C26	121.4 (10)	C80—C81—H81	120.2
C24—C25—H25	119.3	C82—C81—H81	120.2
C26—C25—H25	119.3	C83—C82—C81	118.0 (12)
C27—C26—C25	121.5 (12)	C83—C82—C85	120.3 (14)
C27—C26—H26	119.3	C81—C82—C85	121.8 (14)
C25—C26—H26	119.3	C82—C83—C84	123.0 (13)
C26—C27—C28	116.4 (11)	C82—C83—H83	118.5
C26—C27—C30	121.9 (14)	C84—C83—H83	118.5
C28—C27—C30	121.6 (14)	C83—C84—C79	119.2 (12)
C29—C28—C27	122.3 (12)	C83—C84—H84	120.4
C29—C28—H28	118.9	C79—C84—H84	120.4
C27—C28—H28	118.9	C86—C85—C87	116 (2)
C28—C29—C24	119.8 (11)	C86—C85—C88	100.0 (17)
C28—C29—H29	120.1	C87—C85—C88	105.8 (17)
C24—C29—H29	120.1	C86—C85—C82	112.5 (15)
C33—C30—C31	103 (2)	C87—C85—C82	110.9 (15)
C33—C30—C27	106.9 (16)	C88—C85—C82	111.0 (17)
C31—C30—C27	114.4 (15)	C85—C86—H86A	109.5
C33—C30—C32	106.6 (17)	C85—C86—H86B	109.5
C31—C30—C32	112.7 (19)	H86A—C86—H86B	109.5
C27—C30—C32	112.3 (18)	C85—C86—H86C	109.5
C30—C31—H31A	109.5	H86A—C86—H86C	109.5
C30—C31—H31B	109.5	H86B—C86—H86C	109.5
H31A—C31—H31B	109.5	C85—C87—H87A	109.5
C30—C31—H31C	109.5	C85—C87—H87B	109.5
H31A—C31—H31C	109.5	H87A—C87—H87B	109.5
H31B—C31—H31C	109.5	C85—C87—H87C	109.5
C30—C32—H32A	109.5	H87A—C87—H87C	109.5
C30—C32—H32B	109.5	H87B—C87—H87C	109.5
H32A—C32—H32B	109.5	C85—C88—H88A	109.5
C30—C32—H32C	109.5	C85—C88—H88B	109.5
H32A—C32—H32C	109.5	H88A—C88—H88B	109.5
H32B—C32—H32C	109.5	C85—C88—H88C	109.5
C30—C33—H33A	109.5	H88A—C88—H88C	109.5
C30—C33—H33B	109.5	H88B—C88—H88C	109.5

### Hydrogen-bond geometry (Å, °)

**Table:**

*D*—H···*A*	*D*—H	H···*A*	*D*···*A*	*D*—H···*A*
O2W—H2WB···O4^i^	0.82	2.25	2.824 (8)	127.
O2W—H2WA···O5^i^	0.82	1.95	2.759 (8)	167.
O1W—H1WA···O13^ii^	0.82	2.50	2.866 (8)	108.
O1W—H1WB···O10^ii^	0.82	2.01	2.767 (8)	154.

Symmetry codes: (i) *x*, *y*+1, *z*; (ii) *x*, *y*−1, *z*.
